# Comparative Analysis of the Omics Technologies Used to Study Antimonial, Amphotericin B, and Pentamidine Resistance in *Leishmania*

**DOI:** 10.1155/2014/726328

**Published:** 2014-05-12

**Authors:** Gagandeep Kaur, Bhawana Rajput

**Affiliations:** University Avenue, College of Medical, Veterinary and Life Sciences, University of Glasgow, Lanarkshire G12 8Q, UK

## Abstract

Leishmaniasis is a serious threat in developing countries due to its endemic nature and debilitating symptoms. Extensive research and investigations have been carried out to learn about the mechanism of drug resistance in *Leishmania* but results obtained in the laboratory are not in agreement with those obtained from the field. Also the lack of knowledge about the mode of action for a number of drugs makes the study of drug resistance more complex. A major concern in recent times has been regarding the role of parasitic virulence in drug resistance for *Leishmania*. Researchers have employed various techniques to unravel the facts about resistance and virulence in *Leishmania*. With advent of advanced and more specific means of detection, further hints about probable mechanisms of conferring resistance are expected. This review aims to provide a consolidated picture along with a comparative account of the work done so far to study the mechanism of antimony, amphotericin B, and pentamidine resistance using various techniques.

## 1. Leishmaniasis: Incidence, Cause, and Resistance

Leishmaniasis forms the ninth largest disease burden in the world affecting more than 90 countries on every continent except Antarctica and Australia (see http://www.cdc.gov/parasites/leishmaniasis/epi.html). It is an endemic disease with an estimated 12 million patients currently and a global rise of up to 2 million patients annually (see http://www.who.int/leishmaniasis/en/).

The principal cause of the disease is* Leishmania* parasite. On the basis of symptoms, leishmaniasis can be classified into two forms—cutaneous and visceral.* L. donovani*,* L. infantum,* and* L. chagasi* are the causative agents of visceral leishmaniasis (VL). This form of the disease is characterized by fever, weakness, night sweats, hepatomegaly, or splenomegaly and is mainly reported in regions of India, Bangladesh, Sudan, Ethiopia, and Brazil. Cutaneous leishmaniasis (CL), on the other hand, arises as a sore at the site of insect bite which can proceed to a severe form at times. CL can take two forms: diffuse CL or mucocutaneous leishmaniasis. In diffuse CL, skin lesions are widespread on the body that resemble leprosy. Mucocutaneous leishmaniasis begins with ulceration in the nares that proceeds further to nasal septum, pharynx, or larynx. It can eventually lead to remarkable disfigurement in the patient. It is normally reported in Africa, Latin America, and Middle East. The causative agents for CL have been classified as old and new world species as follows [1, 2]:

 old world CL:* L. major, L. tropica, L. (L.) aethiopica, and L. infantum*;
 new world CL:* L. (L.) mexicana, L. (L.) amazonensis, L. braziliensis, L. (V.) panamensis, L. (V.) peruviana, L. (V.) guyanensis, L. (L.) pifanoi, L. (L.) venezuelensis, L. (L.) shawi, and L. (V.) lainsoni*.

Female sand fly acts as a carrier to transmit the disease-causing parasite into the host. Basically, the parasite enters the body of the host in* metacyclic promastigote* form. Thereafter, it transforms and multiplies into* amastigote* form [2]. Over the years, a number of drugs have been employed for the treatment of the disease amongst which antimony (Sb) containing compounds called antimonials are the most preferred drugs worldwide. A brief account about the mechanism of action and mode of administration of these drugs has been presented in Table 1.

The efficiency of these drugs depends upon

- immune status of the host,
- parasite factors,
- drug pharmacokinetics [1, 3].

But despite so many treatment options, “drug resistance,” especially antimonial resistance, is a serious problem associated with* Leishmania* research. The seriousness of the issue can be assessed from the fact that there are regions in the world that have been reported of being completely resistant towards therapeutics. One such region is that of Bihar (India) which has been reported to be unresponsive towards pentavalent antimonial treatment [3]. Moreover, resistant strains for almost all existing drugs can be obtained under laboratory conditions [4–8]. Interestingly, there are instances of varying virulence in* Leishmania* parasite on attainment of drug resistance [9–12]. This supports the view that there exists a relation between virulence and drug resistance in* Leishmania*. Some scientists are of an opinion that drug resistance comes with a fitness cost. However, not much work has been conducted to relate drug resistance with virulence in order to scientifically support this hypothesis [12].

The present paper aims to provide an overall picture of the techniques used so far to study the area of drug resistance in* Leishmania* taking three major drugs (antimonials, amphotericin B, and pentamidine) into account and to highlight the advantages and drawbacks of each. Although miltefosine is being used extensively for leishmaniasis, we have no reports of clinical incidences of resistance in miltefosine [13, 14]. Thus not much is spoken about miltefosine and its drug resistance in this review.

## 2. Antimonial Resistance in* Leishmania*

Pentavalent antimonials (Sb (V)) are the first line of treatment against leishmaniasis. Studies suggest that antimonials inhibit vital metabolic processes like fatty acid oxidation, glycolysis, and energy metabolism [13]. But the exact mode of action for the drug is still unknown. To add to the trouble is the problem of “drug resistance.” Many attempts have been made to study the mechanism of drug resistance in* Leishmania* but the true phenomenon still remains obscure. The major breakthroughs in this field have been discussed in the following paragraphs.

### 2.1. Advances to Study Mechanism of Antimony Resistance

#### 2.1.1. Gene Amplification (Figure 1)

Genomic studies have revealed gene amplification to be one of the major mechanisms of attaining drug resistance in* Leishmania*. Genes that have been found to be amplified are* MRPA* and* gsh1* [15, 16] and the genomic results have been confirmed through various proteome analyses studies (RT-PCR, and SILAC) as well [17–19].* MRPA* gene codes for an ABC transporter that helps in sequestration of metal-thiol complex in an intracellular organelle which ultimately leads to removal of drug molecules from the cytoplasm [15]. Similarly* gsh1* gene codes for the heavy subunit of *ϒ*-GCS which is a rate-limiting enzyme of glutathione (GSH) biosynthetic pathway that helps in drug detoxification [16].

However, when similar studies were repeated with clinical isolates of* L. donovani*, no noticeable change in expression levels of* MRPA* and* gsh1* genes was established [20]. It was hypothesized that mutation in* Leishmania* parasite is species-specific which results in such contradictions. In recent times, with the advent of next-generation sequencing, this hypothesis could be justified to a certain extent. In fact results obtained from a recent whole genome comparison study conducted on S- and R-strains of* Leishmania* support the fact that the genetic background of the parasitic strain could be the chief reason for the prevalent heterogeneity amongst antimony-resistance phenotypes [21].

#### 2.1.2. Cellular Sequestration or Efflux of Metal-Thiol Conjugate from the Cell (Figure 1)

Genomic comparisons of resistant and sensitive strains of* Leishmania* point towards the overexpression of genes like ODC (ornithine decarboxylase), SAHH, and FPGS in resistant lines [15, 22]. ODC is essential for trypanothione synthesis which is a major thiol for formation of metal-thiol complex thus leading to cellular extrusion of drug molecules [22]. Enhanced levels of thiol metabolising enzymes and oligopeptidase B (*OPB*) have been observed in resistant lines after 2-DE and LC-ES MS/MS comparisons.* OPB* has been found to play a role in immune evasion, fitness, and virulence in* L. donovani* and* L. major* [23]. In fact metabolomic studies performed on resistant strains have also observed manyfold increases in the thiol level of resistant parasite [24].

Studies have proven the importance of ODC overexpression and thiol-conjugated efflux pumps in antimony resistance in various leishmanial strains [17, 25]. A recent study has found a new half-transporter, ABC14, to be involved in antimony resistance. ABC14 has been found to be responsible for the efflux of antimony from antimony-resistant cells of* L. major* in the form of a drug-thiol conjugate [25]. These findings support the role of cellular sequestration and subsequent efflux of drug from the parasitic cytoplasm in the form of metal-thiol conjugate.

#### 2.1.3. Decreased Activation of Antimony Prodrug (Figure 1)

It is not known whether activation of antimony prodrug, that is, conversion of Sb (V) to Sb (III), occurs intracellularly or extracellularly. Yet it is evident that hindrance in activation of Sb prodrug forms the first means of drug resistance in* Leishmania*. An intracellular enzyme, TDR1, has been identified that helps in the conversion of Sb (V) to Sb (III) and is believed to be underexpressed in R-cells [26].

#### 2.1.4. Decreased Uptake of Drug by Underexpression of AQP1 (Figure 1)

Gourbal et al. showed that overexpression of* AQP1* (aquaglyceroporin) gene in resistant strains of* L. tarentolae*,* L. infantum*, and* L. major* results in increased sensitivity of the cells towards trivalent antimonial (Sb (III)) drug [27]. Various studies conducted in recent times confirm these findings [17, 18]. It is thus known that downregulation of* AQP1* plays a significant role in antimony resistance for* Leishmania* as it prevents the entry of Sb (III) into the parasitic cell.

*Recent Findings. *Recently certain new mechanisms like decreased DNA fragmentation and programmed cell death have been identified to confer Sb resistance in* Leishmania* [20].

Additionally sequencing studies are revealing newer mechanisms of resistance. For instance, a novel terminal deletion of chromosome 31 has been reported recently amongst three antimony-resistant mutants of* L. major* through next-generation sequencing and comparative genomic hybridization (CGH) [28].

Comparative proteome analysis of R- and S-strains of* Leishmania *has been conducted in various studies. These studies revealed overexpression of metabolically significant proteins like carboxypeptidase, enolase, fructose-1, 6-bisphosphatase,* Hsp70,* and* Hsp83 *and downregulation of a kinetoplast membrane protein (*KMP11*) and calcineurin in R-strains [29–31].

*Hsp70* has already been known to have an immune-stimulatory role in* L. infantum* and demands further research to indicate its importance in drug resistance. Proteome comparison, DNA fragmentation assays, and mitochondrial membrane potential analysis of two strains of* L. donovani* demonstrated overexpression of* Hsp83* and reduction in DNA fragmentation and membrane potential of resistant parasites [20, 29].

*KMP11* belongs to a highly conserved family of membrane glycoproteins which are said to define virulence in* Leishmania *[30].

Downregulation of calcineurin has been reported for the first time in literature. It is a calcium dependent protein phosphatase involved in varied cellular activities like cell survival and apoptosis. It is thus believed that underexpression of calcineurin can protect resistant parasites from antimony-induced apoptosis [31], although further work needs to be done in this area.

Full metabolome comparisons of sensitive and resistant clinical isolates of* L. donovani* have identified a number of variations between the two phenotypes [32–34]. Major difference has been found in the lipid composition of the two phenotypes. Decreased level of sphingolipids and sphingomyelins and increased levels of phosphatidylcholine and unsaturated fatty acids were observed for resistant strain which indicates involvement of changed membrane fluidity and characteristics for conferring Sb resistance [32].

Levels of surface glycoconjugates were found to be enhanced in R-strains. These glycoconjugates have been known to play some role in metacyclogenesis and virulence of the parasite [33].

A recent metabolomics comparison between resistant and sensitive strains of* L. infantum* promastigotes using CE-ESI-TOF-MS exhibited variations in levels of various amino acids, which were not shown in previous studies [35]. Another study showed the increase in metabolites involved in urea cycle and cysteine transsulfuration pathways [34]. Such results are thus encouraging for the future of drug resistance studies and demand further exploration in this area with the help of modern analytical techniques.

## 3. Amphotericin B Resistance in* Leishmania*

Amphotericin B (AmB) is the second line of defence against leishmaniasis [36]. This polyene drug has high affinity towards membrane-bound ergosterol which leads to the formation of small membranous pores that alters membrane permeability towards cations, water, and glucose molecules [5]. Consequently, osmotic integrity of the cell is disrupted causing leakage of magnesium and potassium ions that finally leads to cell death [37].

AmB resistance in field or clinical isolates is not common for* Leishmania*. Several studies prove instead that AmB susceptibility remains unaffected even after repeated administration of the drug [38, 39]. Although for patients with HIV/VL coinfections the relapse rate after AmB treatment is quite high which might eventually lead to resistance [39]. A recent report from India has identified a patient infected by AmB resistant strain of* L. donovani* which presents the possibility of emergence of other such cases in future [40].

### 3.1. Advances to Study Mechanism of AmB Resistance

#### 3.1.1. Change in Membrane Fluidity (Figure 2)

AmB toxicity relies on the interaction of the drug with parasitic membrane. Hence preliminary studies about AmB resistance relied on flow cytometric assays to investigate membrane potential of* Leishmania* cells [39]. Mbongo et al. (1997) used MS analysis to prove that AmB-resistant promastigotes of* L. donovani* are rich in cholesta-5,7,24-trien-3*β*-ol (a precursor in ergosterol biosynthesis) which results in increased membrane fluidity. Furthermore the intracellular AmB concentration was found to be low for resistant cells [5]. GC-MS studies for sterol analysis proved abundance of methylcholesta sterol in resistant promastigotes and amastigotes under* in vitro* conditions. Interestingly* in vivo* studies performed with the wild-type and drug-resistant amastigotes and promastigotes revealed decreased infectivity in drug-resistant promastigotes and inability to cause infection in resistant amastigotes [4]. The exact reason for this decreased infectivity is not known, but it is reasoned out that decreased microviscosity of membrane in resistant parasites might render membrane receptors nonfunctional which affects the infectivity [5].

#### 3.1.2. Decrease in Thiol and ROS Levels and Increase in Drug Efflux from the Cell (Figure 2)

Recently the mechanism of AmB resistance in clinical isolates of* L. donovani* was studied where an increase in membrane fluidity and decrease in potassium leakage were observed for R-strains. The intracellular thiol and ROS (reactive oxygen species) level was low in resistant cells which proved that tryparedoxin cascade might be operative to prevent oxidative damage by elimination of toxic peroxides from the cells [41].

Furthermore, RT-PCR results of the clinical isolates of AmB-resistant strains showed the upregulation of genes involved in trypanothione biosynthesis and tryparedoxin cascade. The mRNA level of MDR1 (ABC transporter) was found to be 4-fold higher in R-strains which supported the observations for increased drug efflux. Finally the expression of SCMT, an enzyme responsible for C24 transmethylation in sterol biosynthetic pathway, was tested. It has two transcripts—SCMT A and SCMT B. Expression profiling showed lack of expression for SCMT A and increased expression for SCMT B. Altered expression of* SCMT* genes in case of resistance supported the view that defective expression of enzymes involved in sterol biosynthetic pathway leads to absence of membrane ergosterol in R-cells [41]. Further studies on SCMT and other enzymes involved in sterol biosynthesis could be expected to provide some results regarding drug resistance.

#### 3.1.3. Gene Amplification in Extrachromosomal Circle (Figure 2)

An alternative mechanism for drug resistance was tested in* L. tarentolae*. Gene amplification studies for resistance identified two strains exhibiting amplification in extrachromosomal DNA of the parasite. The* in vitro* resistance levels were found to be directly proportional to the copy number of amplicons and the resistance achieved was highly stable [42]. However, the exact region undergoing amplification still remains unknown.

SDS-PAGE results of a recent case report from India revealed overexpression of proteins in the range of 65 to 80 kDa, in AmB-resistant sample. This band was recognised to be cysteine protease. But no known mechanistic significance of this protein has been reported for drug resistance in* Leishmania* to date [40]. Again this is just a case report and this data cannot be completely relied upon as it could be just an individualistic observation.

## 4. Pentamidine Resistance in* Leishmania*

Pentamidine (PMD) has been used as an alternative treatment for VL in cases of Sb resistance. Its exact mode of action is unknown but reports show that it inhibits activity of enzyme S-adenosyl-L-methionine decarboxylase, interferes with polyamine synthesis, and decreases mitochondrial membrane potential [43]. Thus the main target for the drug seems to be parasitic mitochondria [44]. Evidence of resistance against PMD has been reported but the mechanism of resistance is not understood properly [45, 46].

### 4.1. Advances to Study Mechanism of Pentamidine Resistance

#### 4.1.1. Change in kDNA Genome Sequence (Figure 3)

Early experiments using molecular modelling, biophysical analysis, and molecular biology revealed the interaction of PMD with minor groove of AT-rich regions of DNA, that is, kDNA. Hence initial attempts about PMD resistance in* Leishmania* focused on kDNA comparisons. Southern blot comparisons between the kDNA of wild-type and PMD-resistant cells of* L. donovani* and* L. amazonensis* proved significant variations. Sequence homology result was very low (32–51%) thus supporting the view about changes in kDNA sequence to confer resistance in these species [8].

#### 4.1.2. Drug Efflux Pumps (Figure 3)

In a study performed with* L. major*, role of a new gene named* PRP1*, coding for an ABC transporter, was identified in drug resistance [47].* In vitro* study performed on* L. infantum* exhibited that* PRP1*-transfected parasites possessed 3-fold resistance against PMD. Similar results were obtained for* L. mexicana* and* L. amazonensis* [44]. Hence it was confirmed that PRP1 plays a definite role for PMD resistance in* Leishmania*. Presence of other transporters having similar function is being hypothesized but none have yet been recognized [7].

#### 4.1.3. Decreased Mitochondrial Uptake (Figure 3)

Pentamidine accumulation in mitochondria is a reason for its toxicity to parasitic cells. Drug uptake studies showed that the mitochondrial uptake of pentamidine is inhibited in resistant cells which lead to rapid removal of the drug present in the cytosol [48]. Another study performed on* L. amazonensis* indicated modified uptake of spermidine and putrescine in PMD-resistant parasites. Pentamidine uptake by resistant clones was shown to be carrier mediated and energy dependent [43, 49]. The involvement of polyamine biosynthetic pathway in pentamidine resistance was further studied and it was found that intracellular ornithine and arginine levels increase while those of putrescine decrease for PMD-resistant* L. donovani* and* L. amazonensis* cells [49]. It has been known that pentamidine competitively inhibits arginine transport and noncompetitively inhibits spermidine and putrescine transport. Hence increased arginine level and decreased putrescine level are a mechanism to prevent the uptake of pentamidine by the mitochondria. Resistant cells show lowered mitochondrial membrane potential which again help in drug exclusion. Biochemical studies have indicated reduced mitochondrial uptake of PMD in resistant cells. It has been shown that administration of mitochondrial metabolic inhibitors like sodium azide and KCN lowered the PMD uptake thus highlighting its significance as resistance mechanism. However results with P-glycoprotein pump inhibitors (verapamil) varied. While, on one hand, verapamil reversed PMD resistance phenotype for* L. mexicana*, on the other hand no such effect was shown for* L. donovani* [50]. This proves that drug resistance in* Leishmania* is affected by species factors as well.

Various enzyme assays have proved that the levels of enzyme involved in polyamine biosynthesis are altered in PMD-resistant parasites. The level of ODC (ornithine decarboxylase), which converts ornithine to putrescine, is lowered in PMD-resistant cells which accounts for the decreased levels of putrescine in resistant cells [51]. The activity of most enzymes involved in maintenance of mitochondrial potential has also been found to be diminished in R-strains thus leading to lowered membrane potential [50].

## 5. Virulence and Drug Resistance: Are They Related?

“Virulence,” “fitness,” and “parasitic proficiency” are different terms used to describe the set of characteristics present in a parasite to divide and transmit disease. Studies on viruses have pointed towards a decrease in viral replication and differentiation on attaining resistance towards antiretroviral drugs. A similar observation is expected for* Leishmania* as well [12]. In fact, numerous* in vitro* studies prove this thought to be true.

In a report about antimony resistance in American species of* Leishmania* (*L. V. guyanensis*,* L. L. amazonensis*, and* L. V. Braziliensis*) it was seen that resistant parasites had reduced infectivity as compared to their progenitor [52]. Similar results are available in ricin-resistant* L. major*, glucantime-resistant* L. (V) guyanensis,* and AmB-resistant* L. mexicana* [12]. However after subsequent passages in drug absence, the resistant strains were found to be able to grow faster which proved the existence of some compensatory mutation in resistant strains. Thus this led towards deduction of a hypothesis that drug-resistant parasite is likely to be less virulent than wild type. Consequently it was believed that fitness cost can be used as a means to fight leishmaniasis [52]. However,* in vivo* models displayed a different picture altogether. While hypothesizing about decreased fitness of resistant leishmaniasis cells, an important fact about the simplicity of* in vitro* models was neglected. In reality drug resistance is not so simple and is rather achieved via a complex interplay of immune response, nutrient availability, and oxidative stress imposed by environment on* Leishmania*.

*Leishmania* is a highly adaptive parasite. Over thousands of years, it has developed mechanisms to overcome the hostile environment encountered in insect's midgut and host's macrophages [11]. Thus it is not hard to believe that it can develop drug resistance without paying a fitness cost in return. In a study relating metacyclogenesis (parameter for infectivity) to antimony resistance in* L. donovani* it was established that capacity of differentiation was significantly higher in antimony-resistant cells [10]. Another report about clinical isolates taken from Nepalese strains of* L. donovani* showed that resistant cells had the ability to attain a higher parasite density, contained a higher number of metacyclics, and possessed increased capacity of* in vivo* infection than sensitive cells. [11]. Further work done by the same group of researchers successfully proved that resistant strains of* L. donovani* have higher virulence and disease burden as compared to sensitive ones with the help of* in vivo* models [53]. These reports prove that resistance mechanisms derived in the field are quite different from the one observed under laboratory conditions [54]. Moreover it even questions the authenticity of the research done so far regarding drug resistance, as most of the studies are conducted under* in vitro* conditions.

The other question which lingers in mind is about the cause of the increased infectivity in field isolates. A hint about the probable reason was provided during metabolomics comparison between clinical isolates of resistant and sensitive* L. donovani* parasites. This group observed an increase in the amount of surface glycoconjugates in resistant strain. It is known that in* Leishmania* glycosylated proteins and proteoglycans are associated with metacyclogenesis and thus in turn with virulence [33]. So the question arises, is this increase in surface glycosylation a means to improve cell proficiency? And this is not a solo report as it has been stated that changes in metabolic pathways involving pteridine reductase and trypanothione reductase play a role in attaining resistance in* Leishmania*. These have an intimate relationship with parasite's virulence and can be considered to play a specific role in affecting infectivity. Hence it could be considered that such metabolic parameters might be able to predict changes in cell proficiency associated with drug resistance. However, the true nature of this association is still unknown [12].

These findings about increased infectivity of drug-resistant* Leishmania* parasites are an alarm signal. To date we have studied the two phenomena—virulence and drug resistance—separately for* Leishmania*. But recent reports prove the close association between the two. It is thus necessary to design future studies to predict effects of drug resistance in virulence for* Leishmania* under* in vivo* conditions.

## 6. Concluding Remarks and Future Prospects

Drug resistance is a highly complex mechanism and holds a strong association with parasitic infectivity. Contrary to* in vitro* results, field isolates show increased virulence and carry a potential risk of selection of virulent pathogens through chemotherapeutic interventions. The three branches of biotechnology that have been used to date for the study of drug resistance are genomics, proteomics, and metabolomics. A comparative account of these techniques is given in Table 2.

Comparison of complete proteome and metabolome has been employed quite recently. But genomic analysis has been prevalent for a long time. Genomic data is able to provide evidence for the root cause of drug resistance phenotype in* Leishmania*. Also phylogenetic classification of parasitic species helps in prediction of gene function in related species. As such, out of 20 causative species of leishmaniasis only 7 are fully sequenced [55]. Comparison of the genomes of these species reveals high percentage identity. For instance, the average nucleotide identity between* L. major* and* L. infantum* is 94% (*L. major* and* L. braziliensis* is 77% and* L. infantum* and* L. braziliensis* is 77%) [56]. But despite such high level of identity, the pathogenesis of each varies drastically. This either means that pathogenesis in* Leishmania* is determined by just a few species-specific genes or else genome plays a minor role in determining clinical outcome of the disease. Furthermore the sequence information obtained using Sanger sequencing does not provide the knowledge about gene copy number. Use of novel sequencing methods can overcome this shortcoming but at present this lack of data does not work in favour of the researchers [57].

Proteomics, on the other hand, focuses on the functional output of the cell. Essentially, it has been observed that most of the regulation in* Leishmania* is obtained posttranslationally. Hence proteomic studies provide more knowledge about differences between resistant and sensitive parasites as compared to genomic data [58]. It is a good indicator about protein abundance and expression and can yield an insight into species differences, stage differentiation, drug resistance, and virulence [59]. However, on the downside, a number of differentially expressed proteins found after proteome comparison lack an annotated function [57]. Most of the gel-based proteome comparisons only highlight abundant proteins, whereas the not-so-abundant ones seem to be masked [58]. Hence further research needs to be done in characterizing unidentified proteins, not-so-abundant proteins, and proteins with unknown functions.

With the advent of highly specific detection methods, like LC-MS, Orbitrap, GC-MS, and so forth, metabolomics data appears to be highly beneficial for* Leishmania* research. Metabolic profiles can give closest correlation to the phenotype in organisms. Metabolic maps provide a rapid and efficient method to visualize the metabolic changes and consequently predict its biological impact. Also due to high mass accuracy of metabolite masses obtained using recent techniques,* ab initio* extension of metabolic networks is possible. This can be used to make hypothetical connections and predict metabolic transformations related to observed mass peaks. But despite these advantages, still a lot of advancement is required in this field. The current use of LC-MS does not allow efficient quantification of all detected metabolites. Many of the signals thus obtained correspond to analyte derivatives like isotopes, fragments, adducts, and so forth. Biologically this holds no significance and needs to be filtered. Mass spectrometry results generate a large volume of data which cannot be handled and interpreted using the available bioinformatics tools. Thus newer and complicated database and bioinformatics tools need to be developed for handling, filtering, and processing the data with ease [57]. From a personal observation, it can be added though that studies based on two or more strategies are more informative than the ones based on a single strategy.

In the end it could be concluded that drug resistance in leishmaniasis is a major concern for doctors and researchers worldwide. Much work has been done to understand the mechanism of resistance for major drugs against* Leishmania* but not much attempt has been made to study virulence in drug resistance. In recent times this has grabbed the attention of the scientists and further work in this area might reveal significant facts. Genomic, proteomic, and metabolomic approaches used so far have been quite informative. Additionally with advancements in technology better and more effective means for detection and characterization of genes, proteins, and metabolites are available. It could thus be believed that, with combined effort in this area, we might manage to formulate newer drugs, identify good biomarkers, and develop effective vaccines to fight against this disease and eventually overcome the problem of drug resistance which is prevalent at present.

## Figures and Tables

**Figure 1:**
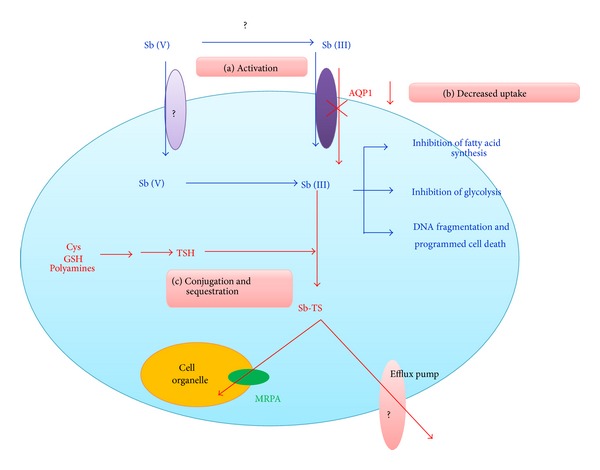
The mechanisms of antimony resistance in* Leishmania*. (a) Activation: conversion of Sb (V) to Sb (III) is inhibited in R-cells. This inhibition can either occur extracellularly (by inactivation of enzymes still unknown) or intracellularly (by inhibition of enzymes like ACR2 or TDR1). (b) Decreased uptake: decreased expression of AQP1 reduces Sb uptake into the cell thus conferring resistance. (c) Conjugation and sequestration: increased thiol levels (like cysteine (Cys), GSH, TSH, and polyamines) in the cell result in its conjugation with antimony to form antimony-thiol complex (Sb-TS) which then results in sequestration of the complex into a cell organelle or extrusion from the cell thus lowering intracellular amount of antimony. Blue lines indicate the probable drug action in sensitive* Leishmania* strains while red lines depict the probable routes to achieve resistance as observed in resistant cells.

**Figure 2:**
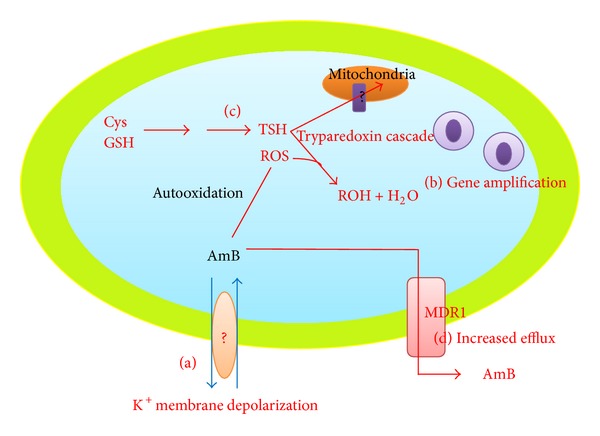
The mechanisms of amphotericin B resistance in* Leishmania. *(a) Change in membrane fluidity results in blocking of the drug entry inside the cell. The membrane transporters or factors responsible for such changes in membrane depolarization are still unknown. (b) Gene amplification of genes to confer resistance. (c) Activation of tryparedoxin cascade to prevent the oxidative damage caused by the drug. (d) Drug efflux through various membrane-bound pumps like MDR1.

**Figure 3:**
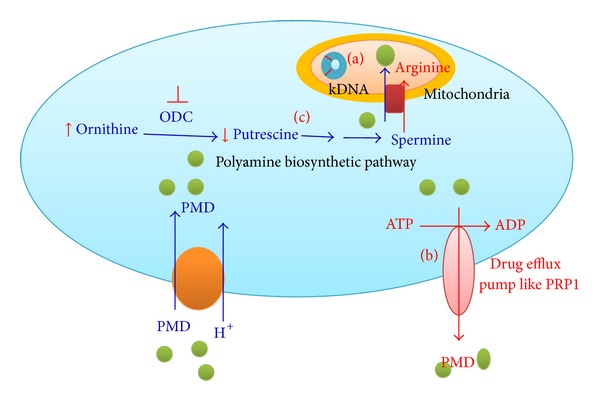
The mechanisms of pentamidine resistance in* Leishmania*. (a) Change in kDNA sequence confers resistance. The exact mechanism of conferring resistance remains unknown. (b) Presence of drug efflux pumps like PRP1 to remove the drug molecules from the cell and thus protect it from damage. (c) Reduced uptake of pentamidine in mitochondria due to altered polyamine biosynthetic pathways and lowered membrane potential. Blue lines indicate the probable drug action in sensitive* Leishmania* strains while red lines depict the probable routes to achieve resistance as observed in resistant cells.

**Table 1:** Drugs used for the treatment of leishmaniasis.

Serial number	Name of the drug	Mode of action	Mode of administration	Adverse effects	References
1	Pentavalent antimonials	Inhibition of glycolysis and *β*-oxidation of fatty acids of parasite	Intralesional for CL Parenteral	Abdominal pain, erythema, nausea, toxicity (hepatic, pancreas, renal, muscular, and skeletal cardiothrombocytopenia or leukopenia)	[2, 60, 61]

2	Amphotericin B	Binding to parasite's membrane sterols and changing its permeability selective to K^+^ and Mg2^+^	Liposomal formulations Deoxycholate formulations	Fever, nausea, hypokalemia, anorexia, leukopenia, kidney failure, and heart problems	[2, 59–61]

3	Pentamidine	Interferes with DNA synthesis and modifies the morphology of kinetoplast	Parenteral Intramuscular administration	Pain, nausea, vomiting, dizziness, myalgia, hypertension, headache, hypoglycemia, and transient hyperglycemia	[1, 2, 60, 61]

4	Miltefosine	Associated with phospholipid biosynthesis and alkyl-lipid metabolism in *Leishmania *	Oral for VL	Nausea, vomiting, diarrhea, and raised creatinine	[1, 2, 59, 60]

5	Paromomycin	Inhibition of protein biosynthesis in sensitive organism	Topical for CL Parenteral for VL	Erythema, pain, oedema, and ototoxicity (damage to internal ear)	[1, 2, 60]

**Table 2:** The advantages and disadvantages of strategies used to study drug resistance.

	Techniques	Advantages	Disadvantages	References
Genomics	Southern blot, microarray, northern blot, sequencing, PFGE, FIGE	(a) Genetic basis of observed phenotype (b) Large genome coverage (22–97.5%) (c) Easy to perform and interpret (d) Reproducible	(a) Not much differences amongst species at genome level (b) Not much informative (c) Gene functions for most genes still unknown.	[7, 8, 14, 15, 22, 37, 41, 42, 44, 47, 51, 62]

Proteomics	2DE, MALDI-TOF, LC-MS/MS, LC-ESI-MS/MS, western blot, immunoblot	(a) Functional output of the cell (b) Posttranslational changes visualised (c) Mostly automated (d) Good indicator of protein abundance and expression	(a) Number of proteins lack annotated functions (b) Less abundant proteins hard to detect (c) Results from *in vivo* amastigotes difficult to interpret (d) Not reproducible in some cases	[6, 20, 23, 29, 30, 40, 49, 51, 51]

Metabolomics	CE-ESI-TOF-MS, HPLC, MALDI-TOF, flow cytometry, GC-MS	(a) Closest correlation to phenotype (b) Rapid visualisation and prediction of biological impact (c) High mass accuracy (d) Highly specific	(a) Unable to quantify most of the metabolites. (b) Analyte derivatives make data complex (c) Data hard to analyse (d) Complicated bioinformatics tools needed (e) Costly instrumentation	[4, 5, 22, 24, 32, 35, 41, 43, 48, 51]

PFGE: pulsed field gel electrophoresis; FIGE: field inversion gel electrophoresis; 2DE: two-dimensional gel electrophoresis; MALDI-TOF MS: matrix assisted laser desorption/ionisation time of flight mass spectrometry; LC-MS/MS: liquid chromatography mass spectra/mass spectrometry; LC-ESI-MS/MS: liquid chromatography electrospray ionisation tandem mass spectrometry; CE-ESI-TOF-MS: capillary electrophoresis mass spectrometry coupled with electrospray ionisation mass spectrometry; HPLC: high performance liquid chromatography; GC-MS: gas chromatography mass spectrometry.

## Glossary

*AQP1* gene: Aquaglyceroporin gene
Drug resistance: Reduction in effectiveness of a drug in curing a disease or condition
GC-MS: Gas chromatography-mass spectrometry
GSH: Glutathione
*GSH1* gene: Gamma-glutamylcysteine synthetase gene
kDa: Kilo Dalton
kDNA: Kinetoplastid deoxyribonucleic acid
MDR1: Multidrug-resistant protein 1
Metacyclogenesis: The differentiation of metacyclic promastigotes
Microviscosity: Friction experienced by a single particle undergoing diffusion because of its interaction with its environment at the micrometre length scale
Pharmacokinetics: Branch of pharmacology concerned with the movement of drugs within the body
Prodrug: A biologically inactive compound that can be metabolized in the body to produce a drug
PRP1: Pentamidine resistance protein 1
Relapse rate: The rate of reemergence of disease symptoms in a patient after treatment
SCMT: S-Adenosyl-l-methionine: C-24-delta-sterol-methyltransferase
Sequence homology: Sequence similarity as a result of common ancestry
TDR1 enzyme: Thiol dependent reductase.
